# Multimodal Anesthetic Strategy to Facilitate Early Extubation in a Patient With Severe Restrictive Lung Disease Undergoing Major Scoliosis Surgery: A Case Report

**DOI:** 10.7759/cureus.106897

**Published:** 2026-04-12

**Authors:** Mohammed I Hakami, Omimah T Barnawi

**Affiliations:** 1 Anesthesiology, King Faisal Specialist Hospital and Research Centre, Riyadh, SAU

**Keywords:** anesthetic management, intravenous lidocaine infusion, iv ketamine infusion, multimodal analgesic, restrictive lung disorder, scoliosis surgery

## Abstract

Posterior scoliosis correction is associated with severe postoperative pain and substantial opioid requirements, which may pose a significant risk in patients with limited ventilatory reserve. We report the anesthetic management of a 20-year-old male with extremely severe scoliosis and very severe restrictive lung disease undergoing prolonged multilevel posterior spinal fusion with intraoperative neuromonitoring. A multimodal total intravenous anesthetic technique incorporating ketamine and lidocaine infusions was employed. This approach was associated with the preservation of neuromonitoring signals, successful end-of-case extubation in the operating room to supplemental oxygen via simple face mask, and low postoperative opioid requirements. This case highlights the importance of opioid minimization when managing high-risk patients with compromised pulmonary reserve.

## Introduction

Posterior scoliosis correction is one of the most painful orthopedic procedures due to extensive muscle dissection, osteotomies, and spinal instrumentation. Postoperative analgesia has traditionally relied on opioids, with reported requirements ranging from 55 to 180 mg oral morphine equivalents per day [1]. However, in patients with severely limited pulmonary reserve, opioid-induced ventilatory depression may pose a significant risk.

Severe scoliosis is frequently associated with restrictive lung disease, resulting in limited ventilatory reserve and increased susceptibility to postoperative respiratory complications, delayed extubation, and prolonged intensive care unit (ICU) stays [2]. In such patients, anesthetic management requires a careful balance between adequate analgesia and preservation of respiratory function.

Multimodal anesthetic strategies incorporating non-opioid adjuncts have been increasingly adopted within enhanced recovery pathways to reduce opioid exposure while maintaining analgesic efficacy [3,4]. Among these, ketamine and lidocaine infusions have demonstrated analgesic and opioid-sparing effects, with minimal interference on intraoperative neuromonitoring signals [5-7]. However, evidence supporting their combined use specifically to facilitate early extubation in patients with severe restrictive lung disease undergoing major spine surgery remains limited.

We report the anesthetic management of a young adult with extremely severe scoliosis and very severe restrictive lung disease undergoing prolonged multilevel posterior spinal fusion. In this report, “early extubation” refers to successful end-of-case extubation in the operating room rather than planned postoperative ventilation. The peri-extubation course is described using serial arterial blood gas measurements, immediate post-extubation oxygen requirements, and postoperative opioid consumption. This case highlights the possible role of an opioid-sparing total intravenous anesthesia (TIVA) technique incorporating ketamine and lidocaine infusions in achieving successful end-of-case extubation, preserving neuromonitoring signals, and maintaining low postoperative opioid requirements in a high-risk patient.

## Case presentation

A 20-year-old male (weight 49 kg) with progressive idiopathic scoliosis diagnosed at 16 years of age presented for elective posterior spinal fusion. He reported worsening dyspnea on exertion. Pulmonary function testing demonstrated very severe restrictive lung disease with a forced vital capacity (FVC) of 32% predicted and a forced expiratory volume in one second (FEV₁) of 27% predicted (Table 1).

**Table 1:** Preoperative pulmonary function test Findings are consistent with severe restrictive lung disease. FVC: forced vital capacity; FEV₁: forced expiratory volume in one second; FEF: forced expiratory flow

Parameter	Actual	Predicted	% Predicted
FVC (L)	1.26	3.91	32%
FEV1 (L)	0.93	3.41	27%
FEV1/FVC (%)	74	85	86%
FEF 25% (L/s)	2.67	8.04	33%
FEF 75% (L/s)	0.18	3.52	4%
FEF 25-75% (L/s)	0.65	3.89	16%
FEF Max (L/s)	2.77	7.40	37%

**Figure 1:**
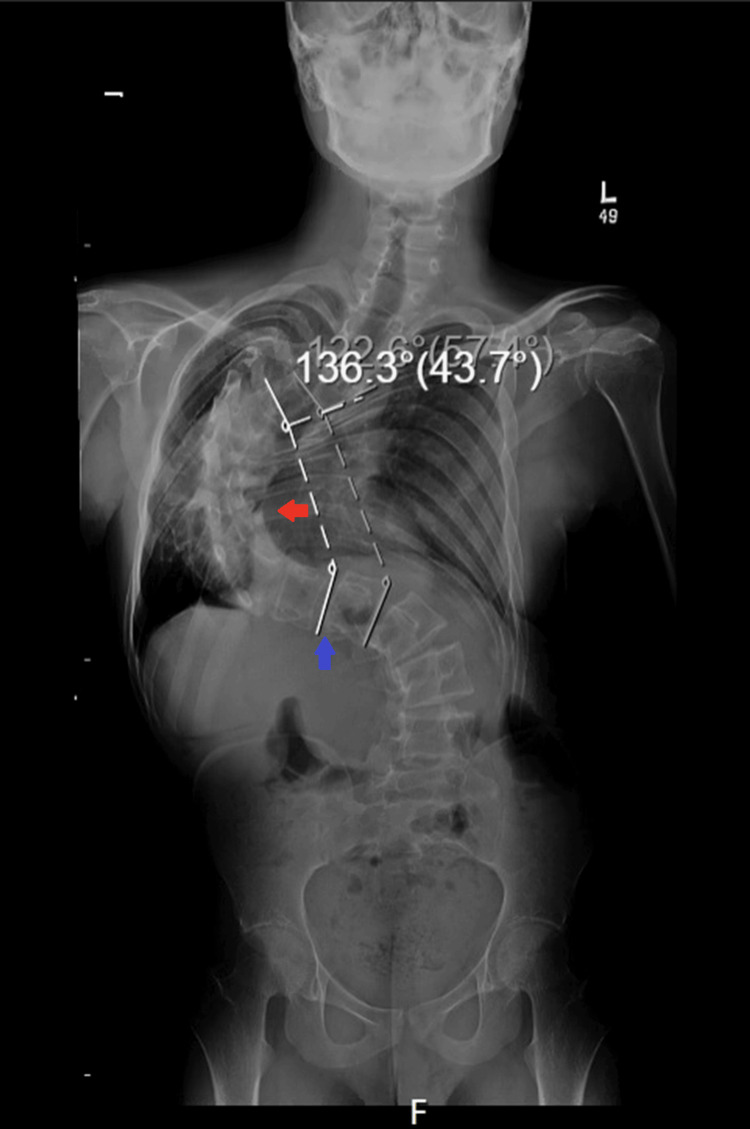
Preoperative thoracolumbar anteroposterior radiograph Preoperative anteroposterior thoracolumbar radiograph demonstrating severe scoliosis with a Cobb angle of 136°. The red arrow indicates the apex of the thoracic curve, while the blue arrow indicates the apex of the compensatory lumbar curve.

Preoperative transthoracic echocardiography, based on the formal cardiology report, demonstrated normal biventricular function. Preoperative thoracolumbar radiography demonstrated a severe right-sided thoracic scoliosis with a Cobb angle of approximately 136° (Figure 1).

Baseline arterial blood gas analysis on room air demonstrated compensated respiratory status (pH 7.38, pCO₂ 5.70 kPa, pO₂ 9.0 kPa). Other laboratory investigations, including renal function tests, electrolytes, liver function tests, and complete blood count, were within normal limits.

The patient had no significant comorbidities and no history of chronic opioid use. On the day of surgery, standard American Society of Anesthesiologists monitors were applied in addition to invasive arterial blood pressure monitoring, bispectral index (BIS) monitoring, and intraoperative somatosensory and motor evoked potential (SSEP/MEP) monitoring.

After preoxygenation, general anesthesia was induced with propofol 2 mg·kg⁻¹, fentanyl 2 µg·kg⁻¹, and rocuronium 1 mg·kg⁻¹ to facilitate tracheal intubation. Anesthesia was maintained using a propofol infusion, and remifentanil was titrated to maintain a BIS between 40 and 60. Neuromuscular blockade was not continued following induction.

Adjunctive multimodal analgesia included ketamine 0.25 mg·kg⁻¹ and lidocaine 1 mg·kg⁻¹ administered prior to skin incision, followed by continuous infusions of ketamine 0.25 mg·kg⁻¹·h⁻¹ and lidocaine 1 mg·kg⁻¹·h⁻¹ until skin closure. Additional adjuncts included dexamethasone 0.25 mg·kg⁻¹, paracetamol 15 mg·kg⁻¹, ketorolac 0.5 mg·kg⁻¹, and magnesium sulfate 40 mg·kg⁻¹ administered slowly.

This anesthetic regimen provided stable hemodynamics throughout the procedure and preserved SSEP and MEP signals without significant attenuation. Lung-protective ventilation strategies were employed, with normocapnia and normoxia maintained intraoperatively.

Posterior scoliosis correction and instrumented fusion from T1 to L5 were completed over nine hours. Estimated blood loss was approximately 750 mL, and 2 units of cell-saved blood were reinfused.

Approximately 45 minutes before the end of surgery, the patient developed tachypnea during pressure support ventilation, along with increased heart rate and blood pressure despite an adequate depth of anesthesia monitored by BIS. These findings were interpreted as consistent with pain-related sympathetic activation. Morphine 0.1 mg/kg was administered to provide transitional analgesia and reduce excessive respiratory effort at emergence.

During emergence, the patient initially demonstrated adequate inspiratory effort. Several minutes later, hypoventilation developed with respiratory acidosis confirmed on arterial blood gas analysis (pH 7.14, pCO₂ 9.50 kPa). Administration of low-dose naloxone (80 µg IV) promptly restored respiratory drive and alertness, with repeat arterial blood gas analysis 10-15 minutes later showing improvement (pH 7.26, pCO₂ 6.30 kPa), without compromising analgesia (Table 2).

**Table 2:** Arterial blood gas values at different perioperative time points Arterial blood gas values at baseline (room air), during hypoventilation prior to naloxone administration (while receiving supplemental oxygen), and after naloxone administration following extubation (on face mask). Values demonstrate acute hypercapnic respiratory acidosis with subsequent improvement after opioid reversal.

Parameter	Baseline (room air)	Pre-naloxone	Post-naloxone
pH	7.38	7.14	7.26
pCO₂ (kPa)	5.70	9.50	6.30
pO₂ (kPa)	9.00	21.70	15.5
HCO₃⁻ (mmol/L)	25.40	24.20	21.10
Base excess	0.1	-6.2	-6.0
Lactate (mmol/L)	0.8	1.2	1.2
SaO₂ (%)	94.2	99.7	98.4

Before extubation, the patient was receiving pressure support ventilation with positive end-expiratory pressure (PEEP) 5 cm H₂O, tidal volumes of approximately 280-300 mL assisted with pressure of 3 cm H₂O, and end-tidal carbon dioxide around 38 mmHg. Extubation readiness was assessed clinically based on adequate spontaneous ventilation, improved arterial blood gas values, hemodynamic stability, and reassuring neurologic examination. Tracheal extubation was subsequently performed uneventfully to 6 L/min supplemental oxygen via simple face mask, with oxygen saturation of 100%, and no pain reported by the patient in the immediate post-extubation period.

He was transferred to the ICU for postoperative monitoring. On arrival to the ICU, he maintained oxygen saturation of 96% on room air. Postoperative analgesia consisted of intravenous morphine patient-controlled analgesia, scheduled paracetamol, and ketorolac. No ketamine or lidocaine infusions were continued postoperatively.

Pain remained well-controlled, and the patient demonstrated early mobilization starting from postoperative day one without significant dyspnea or discomfort. He maintained low opioid requirements throughout hospitalization (Table 3) and was discharged home on postoperative day 5 without complications.

**Table 3:** In-hospital opioid consumption expressed as oral morphine equivalents Oral oxycodone was converted to oral morphine equivalent using a factor of 1.5; IV morphine was converted using a factor of 3.

Postoperative day	Intravenous morphine (mg)	Oral oxycodone (mg)	Oral morphine equivalents (mg)
Day 0	6	0	18
Day 1	2	10	21
Day 2	12	40	96
Day 3	2	30	51
Day 4	0	20	30
Day 5	0	20	30
Total	22	120	246 (41 mg /day)

**Figure 2:**
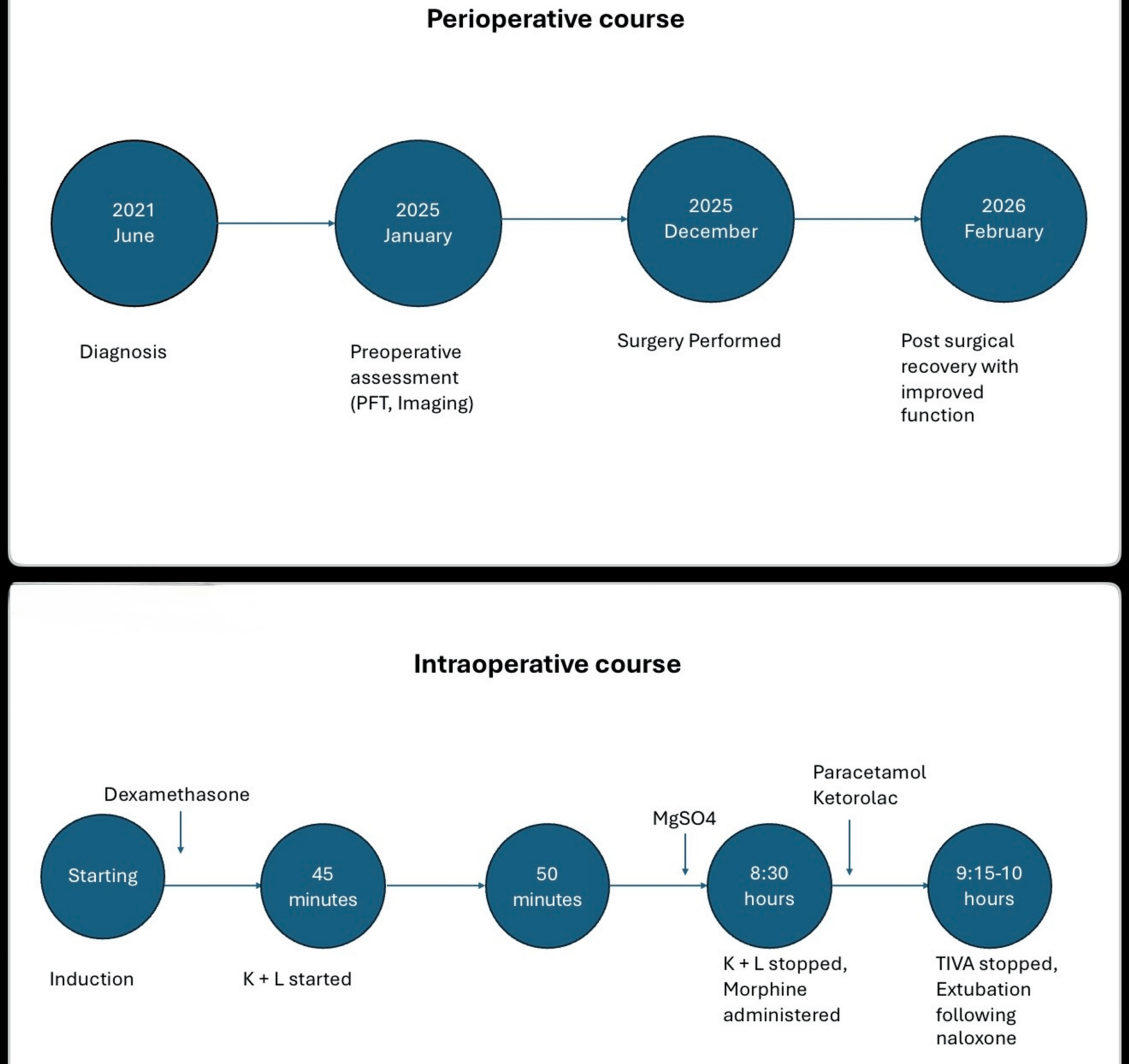
Integrated perioperative and intraoperative timeline of anesthetic management and clinical course The figure illustrates the patient’s perioperative clinical course (top panel) alongside the intraoperative anesthetic timeline (bottom panel). The perioperative timeline summarizes key milestones from initial diagnosis to postoperative recovery. Preoperative evaluation demonstrated severe restrictive lung disease with otherwise unremarkable cardiac assessment. The intraoperative timeline outlines the sequence of anesthetic interventions relative to induction. Following induction and neuromonitoring setup, ketamine (K) 0.25 mg·kg⁻¹ and lidocaine (L) 1 mg·kg⁻¹ boluses were administered, followed by continuous infusions at rates of 0.25 mg·kg⁻¹·h⁻¹ and 1 mg·kg⁻¹·h⁻¹, respectively, initiated at 45 minutes, preceding surgical incision at 50 minutes. Maintenance anesthesia consisted of total intravenous anesthesia with propofol and remifentanil in combination with ketamine and lidocaine, with stable neuromonitoring signals throughout. Midway through the procedure, at approximately the sixth hour, magnesium sulfate 40 mg·kg⁻¹ was administered over 30 minutes. During surgical closure at approximately 8.5 hours, ketamine and lidocaine infusions were discontinued, and adjunct analgesia with ketorolac 0.5 mg·kg⁻¹ and paracetamol 15 mg·kg⁻¹ was administered, along with morphine. This was followed by the development of hypoventilation with respiratory acidosis, necessitating naloxone administration prior to successful extubation at approximately 10 hours after induction. The figure was created using Microsoft PowerPoint (Microsoft Corporation, Redmond, WA, US).

At the eight-week follow-up, he reported improved mobility and posture with minimal pain. A summary of the patient’s perioperative course is illustrated in Figure 2.

## Discussion

This case illustrates several important considerations in the anesthetic management of patients undergoing extensive posterior spinal fusion with coexisting severe restrictive lung disease. In such patients, opioid-induced ventilatory depression poses a significant risk during emergence and may delay or preclude safe extubation.

Unlike volatile anesthetics, which may increase SSEP latency and reduce signal amplitude, propofol-based TIVA has minimal effects on SSEP and relatively modest effects on MEP, making it well-suited for procedures requiring neuromonitoring [5]. Ketamine has been shown to increase evoked potential amplitudes, while lidocaine appears to have minimal impact on neuromonitoring signals, supporting their use as adjuncts during spine surgery [5,6].

Ketamine exerts its analgesic and antihyperalgesic effects primarily through noncompetitive antagonism of the N-methyl-D-aspartate (NMDA) receptor. Lidocaine provides systemic analgesia via blockade of voltage-gated sodium channels and has also been shown to exhibit NMDA receptor-modulating and anti-inflammatory properties. The combination of these agents may result in synergistic or supra-additive effects, contributing to reduced perioperative opioid requirements [7-9].

A key observation in this case is the development of significant respiratory acidosis following the administration of a standard opioid dose, highlighting the vulnerability of patients with severe restrictive lung disease to opioid induced ventilatory depression. Arterial blood gas analysis demonstrated marked hypercapnia (pCO₂ 9.50 kPa) and acidosis (pH 7.14), with subsequent improvement following naloxone administration, supporting an opioid-related mechanism. The rapid restoration of ventilation with low-dose naloxone, without loss of analgesia, suggests that the ketamine- and lidocaine-based multimodal strategy may have helped maintain pain control while limiting overall opioid exposure, while preserving respiratory function.

Although ketamine alone has shown mixed results in reducing postoperative opioid requirements following scoliosis surgery, the combination of ketamine and lidocaine may offer synergistic analgesic benefits. Prior studies have demonstrated reduced opioid consumption and improved pain control when these agents are used together as part of a multimodal analgesic strategy [4,7]. Enhanced recovery protocols often recommend continuation of ketamine and lidocaine infusions into the postoperative period [3,4]. In contrast, in this case, these infusions were discontinued at the end of surgery, yet postoperative pain control remained satisfactory. This suggests that effective intraoperative analgesic optimization may influence postoperative outcomes.

Patients undergoing scoliosis correction with advanced deformity and restrictive lung disease are at increased risk of postoperative pulmonary complications. Risk factors include FEV1 <40%, FVC <39.5%, age >16.5 years, and Cobb angle >69° [2]. Although these thresholds were derived from a neuromuscular scoliosis population, our patient shared similarly severe physiologic and anatomic features, suggesting elevated pulmonary risk. Furthermore, pulmonary function has been shown to decline significantly in the immediate postoperative period following scoliosis surgery [10]. In a large cohort of 9,734 patients undergoing adult spinal deformity surgery, the incidence of reintubation was 1.8%; risk factors of reintubation included pre-existing lung disease and fusion of 8 or more segments. Reintubation was associated with markedly increased mortality as well (7.3% vs 0.2%) [11]. Despite these risk factors, successful end-of-case extubation was achieved safely in our patient. This favorable outcome may have been influenced by the opioid-sparing analgesic strategy.

Total opioid consumption in this patient remained lower than typically reported following multilevel posterior spinal fusion [1]. Early ICU discharge and shortened hospital length of stay compared to prior studies further suggest a potential benefit of this approach [12].

This report has several limitations. As a single case report, causality between the anesthetic strategy and successful early extubation cannot be established. In addition, perioperative outcomes may have been influenced by other factors, including surgical correction, intensive postoperative monitoring, and individualized ventilatory support. Therefore, these findings may not be generalizable to all patients with severe restrictive lung disease undergoing major spine surgery.

## Conclusions

This case highlights the anesthetic challenges associated with managing patients with severe restrictive lung disease undergoing major spinal surgery, particularly in the context of high postoperative opioid requirements and the risk of respiratory compromise. A multimodal total intravenous anesthesia technique incorporating ketamine and lidocaine infusions was associated with preserved neuromonitoring signals, satisfactory analgesia, and successful end-of-case extubation despite limited pulmonary reserve. The episode of opioid-related hypoventilation and its prompt reversal further supports the importance of minimizing opioid burden in this high-risk population. As a single case report, causality cannot be established; however, these findings suggest a possible role for such multimodal strategies in selected patients. Further studies are warranted.
